# Co-occurrence and risk assessment of ochratoxin A and deoxynivalenol in tortillas

**DOI:** 10.1007/s12550-025-00596-z

**Published:** 2025-06-24

**Authors:** Fatma Oznur Afacan, Eylem Odabas, Nimo Hussein Yussuf, Bulent Kabak

**Affiliations:** 1 Department of Nutrition and Dietetics, Faculty of Health Sciences, Selçuk University, Konya, 42130 Türkiye; 2 Department of Food Engineering, Faculty of Engineering, Hitit University, Corum, 19030 Türkiye; 3 Biotechnology Laboratory, Machinery and Manufacturing Technology Application and Research Center, Hitit University, Corum, 19030 Türkiye

**Keywords:** Cereal products, Tortillas, Mycotoxins, Food safety, Dietary exposure, HPLC

## Abstract

The presence of mycotoxins, including ochratoxin A (OTA) and deoxynivalenol (DON), in tortillas raises potential food safety concerns, particularly in regions where these products constitute a dietary staple. In the present study, the natural occurrence and concentrations of OTA and DON were determined in commercially available tortillas in Turkey. A total of 84 tortilla samples were analysed using an in-house validated high-performance liquid chromatography (HPLC) method. OTA was detected in 25% of the samples, with concentrations ranging from 0.079 to 0.93 µg/kg, all of which remained below the European Union maximum level (EU ML) of 3 µg/kg. DON was found in 10.7% of the samples, with concentrations between 13.1 and 158 µg/kg, also within the EU ML of 400 µg/kg. Co-occurrence of OTA and DON was observed in three samples. These findings highlight considered variability among products, likely attributable to differences in raw material quality, environmental conditions, and post-harvest handling practices. This variability underscores the need for enhanced regulatory surveillance, improved storage conditions, and the implementation of optimised processing technologies. Furthermore, the estimated dietary exposure levels for both OTA and DON were found to pose no significant health risks under current consumption patterns.

## Introduction

Tortillas are widely regarded as a staple food in numerous cultures, particularly in Latin America, and are recognized for their significant contribution to global diets. These nutrient-dense flatbreads are traditionally made from maize (*Zea mays* L.) or wheat (*Triticum* spp.), with diameters ranging from 12 to 18 cm and thicknesses between 1 and 4 mm. They have gained popularity due to their versatility and adaptability across various culinary traditions (Astorga-Gaxiola et al. 2023). Their cost-effectiveness and suitability for diverse dietary patterns have further reinforced their indispensable role in both traditional and modern food systems. The popularity of tortillas has grown dramatically over the years, with consumption currently observed across various regions worldwide, including Central America, Europe, and parts of Asia (Cortés-Gómez et al. 2005).

Like other cereal-based products, tortillas are susceptible to mycotoxin contamination during cultivation, processing, and storage. These toxic secondary metabolites, produced by specific fungi, pose significant risks to food safety and public health. Among them, aflatoxins (AFs), fumonisins (FUM), zearalenone (ZEA), ochratoxin A (OTA), and deoxynivalenol (DON) are of particular concern due to their toxicity, stability, and widespread occurrence in cereal-based foods (EFSA 2013; EFSA 2020).

Tortillas are mainly produced from maize or wheat, both of which are susceptible to fungal contamination during pre- and post-harvest stages. The production of maize-based tortillas often involves nixtamalization, a traditional process in which maize kernels are treated with an alkaline solution, enhancing their nutritional value while reducing certain contaminants, including AFs and FUM (Schaarschmidt and Fauhl-Hassek 2019). However, this process does not completely eliminate the risk of contamination by OTA or DON, which may persist in raw materials or develop under inadequate storage conditions. In contrast, wheat tortillas are vulnerable to contamination by *Fusarium* species, which are the primary producers of DON. This mycotoxin is frequently associated with *Fusarium* head blight (FHB), a disease that affects wheat and other small grains under warm and humid conditions. The processing of wheat into flour and the subsequent production of tortillas do not guarantee the elimination of DON, as it is highly stable and resistant to both thermal and chemical degradation (Abbas et al. 1988; Gilbert-Sandoval et al. 2020). In addition, OTA contamination may occur in wheat-based tortillas due to the presence of toxigenic species such as *Penicillium verrucosum*, which thrive under improper storage conditions.

OTA is a nephrotoxic mycotoxin primarily produced by *Aspergillus carbonarius*, *Aspergillus ochraceus*, *Aspergillus westerdijkiae*, and *Penicillium verrucosum*. It exhibits a wide range of toxic effects, including genotoxicity, nephrotoxicity, hepatotoxicity, neurotoxicity, and immunotoxicity (EFSA 2020). OTA has been classified by the International Agency for Research on Cancer (IARC) as possibly carcinogenic to humans (Group 2B) (IARC 1993). Notably, OTA exposure has been associated with Balkan Endemic Nephropathy, a fatal kidney disease reported in certain regions of Eastern Europe. OTA contamination has been documented in a variety of food products, including cereals and cereal-based products such as bread and pasta (EFSA 2020). However, its presence in tortillas has rarely been investigated in the scientific literature, despite the fact that tortillas are considered staple foods in several countries and their consumption has increased significantly in recent years. This limited availability of occurrence data on OTA in tortillas represents a notable knowledge gap, particularly considering the public health implications of chronic exposure to low levels of this toxin.

DON, also known as vomitoxin, is primarily produced by *Fusarium graminearum* and *Fusarium culmorum* (EFSA 2013). It is considered one of the most prevalent mycotoxins globally, particularly affecting cereal crops such as wheat, barley, and maize. In humans, exposure to DON has been associated with acute gastrointestinal symptoms, including nausea, vomiting, diarrhoea, abdominal pain, headaches, and fever (EFSA 2017), while chronic exposure has been linked to immunosuppression and disturbances in gut health (Liao et al. 2018). The high stability of DON during food processing presents significant challenge, as conventional methods such as milling, baking, and cooking are largely ineffective in reducing its concentration. Given its persistence and widespread occurrence in cereals, the presence of DON in tortilla products warrants rigorous monitoring to ensure food safety.

The widespread consumption of tortillas across various regions highlights the importance of managing risks associated with mycotoxin contamination (Wall-Martínez et al. 2019). While extensive research has focused on raw cereal grains, there is a notable lack of studies specifically addressing the presence of OTA and DON in tortillas. This gap is concerning, given the potential for contamination during processing, transportation, and storage. Furthermore, climate change is anticipated to influence fungal growth and mycotoxin production, thereby exacerbating contamination risks in staple foods. Shifts in temperature and humidity patterns may alter both the prevalence and geographical distribution of OTA- and DON-producing fungi.

In light of these concerns, the present study aims to address a critical knowledge gap by investigating the natural occurrence of OTA and DON in commercially available tortillas, and by assessing the associated health risks through chronic dietary exposure estimation, using margin of exposure (MOE) analysis for OTA and comparison with the tolerable daily intake (TDI) for DON. Contamination levels were quantified using validated analytical methods based on high-performance liquid chromatography (HPLC) coupled with fluorescence or ultraviolet detectors (HPLC-FLD or HPLC–UV). To the best of our knowledge, this is among the first studies to simultaneously report OTA and DON occurrence in tortillas alongside a dietary risk assessment. These findings will provide a scientific basis for developing mitigation strategies and informing regulatory actions to improve the safety of tortillas and related cereal-based products.

## Materials and methods

### Reagents, chemicals and standards

HPLC-grade methanol was purchased from Honeywell (Seelze, Germany), and HPLC-grade acetonitrile was obtained from Merck KGaA (Darmstadt, Germany). Ultra-pure water was produced using a Direct-Q3 water purification system (Millipore, Molsheim, France). Glacial acetic acid was supplied by Sigma-Aldrich Chemie GmbH (Steinheim, Germany). Glass microfiber filters (GF/A, 125 mm in diameter) used for filtration during sample preparation were procured from VWR International (Leuven, Germany). For selective clean-up, immunoaffinity columns (IACs) were employed. DONtest™ HPLC IACs (VICAM, Watertown, MA, USA) were used for the analysis of DON, whereas PriboFast® IACs (Pribolab, Qingdao, China) were used for OTA.

The DON standard was obtained from Sigma-Aldrich (St. Louis, MO, USA) in powder form and was reconstituted in methanol to prepare a stock solution at a concentration of 200 μg/mL. This stock solution was subsequently used in spiking in recovery experiments and for the preparation of calibration standards ranging from 10 to 500 μg/L.

The OTA standard (1 mg) was purchased from Pribolab (Qingdao, China), also in powder form. A stock solution containing 100 μg OTA was prepared in methanol and stored at −18 °C. This solution was used for the preparation of calibration standards at concentrations ranging from 0.25 to 20 μg/L in a mobile phase consisting of water–acetonitrile–acetic acid (51:47:2, v/v/v), and for spiking food matrices in the validation study.

### Food samples

**Fig. 1:**
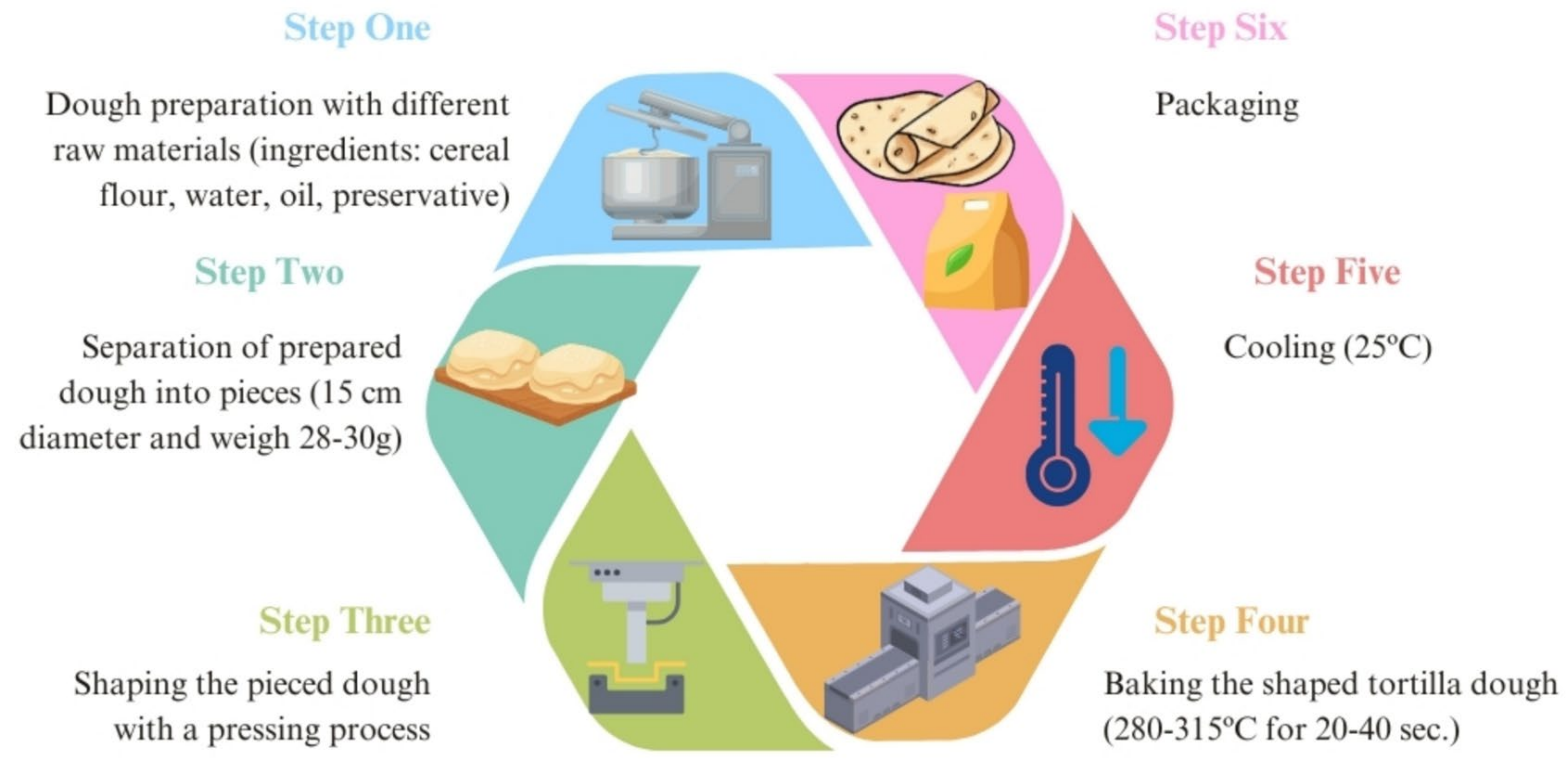
Flow diagram illustrating the manufacturing process of tortilla

A total of 84 tortilla samples were analysed between February and May 2024 to assess the contamination levels of OTA and DON. The samples were randomly collected in their original packaging from tortilla manufacturing companies in Corum Province, Turkey. To ensure product diversity, five distinct brands, designated here as Brand A through Brand E, were included in the sampling. Batch numbers, production dates, and expiration dates were recorded to enhance traceability and reproducibility. Based on label information, the primary ingredients included cereal flour (e.g., wheat, maize, oat), water, salt, leavening agents, and vegetable oils, along with minor additions of emulsifiers, stabilisers, acidity regulators, and preservatives. Slight variations in formulation were observed across different brands and tortilla types, including the presence or absence of additional flavouring components such as chia seeds, chili, chocolate or spinach. The tortilla production process is illustrated in detail in Fig. 1. The sample set included wheat tortillas (*n* = 16), whole wheat tortillas (*n* = 17), whole grain tortillas (*n* = 10), maize flour tortillas (*n* = 13), oat flour tortillas (*n* = 10), and other varieties (*n* = 18). Package sizes ranged from 300 to 650 g. All samples were stored at 4 ± 1 °C and analysed within 1–2 days to minimise the risk of fungal growth. Prior to analysis, each sample was dried at 60 °C in a laboratory oven (Memmert IN110, Büchenbach, Germany). To reduce the potential impact of heterogeneous mycotoxin distribution, samples were homogenised using a Waring blender (Waring Products Co., Connecticut, USA).

### Sample preparation and chromatographic analysis

**Fig. 2:**
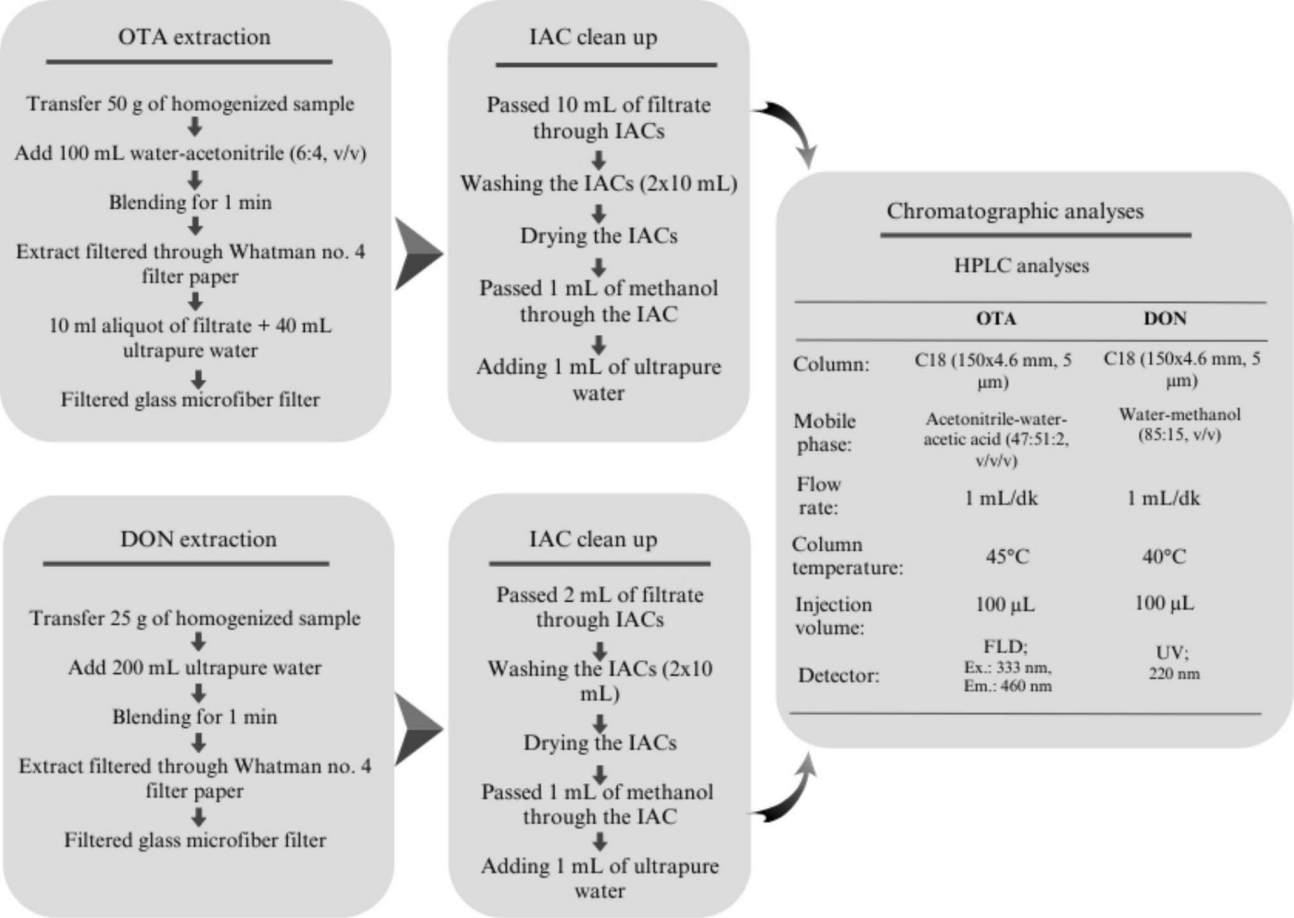
Schematic representation of the analytical workflow for the extraction, IAC purification, and chromatographic determination of OTA and DON

Tortilla samples were processed for OTA and DON analysis using extraction and IAC clean-up methods based on the AOAC Official Method 2000.03 (Entwisle et al. 2000) for OTA and the R-Biopharm guide (R-Biopharm Rhone 2009) for DON, with minor modifications. As illustrated in Fig. 2, 50 g of each homogenized tortilla sample was extracted with 100 mL of acetonitrile–water (6:4, v/v) for OTA, while 25 g of sample was extracted with 200 mL of ultrapure water for DON. Extractions were performed using a Waring blender (Waring Products Co., Connecticut, USA) at high speed for 1 min to ensure efficient mycotoxin recovery.

For OTA, the extract was filtered through pre-folded filter paper (Filtros Anoia, S.A., Barcelona, Spain). A 10 mL portion of the filtrate was diluted with 40 mL of ultrapure water and filtered through a glass microfiber filter. Subsequently, 10 mL of this solution was passed through a PriboFast® OTA IAC from Pribolab (Beijing, China) using a vacuum manifold (Agilent Technologies, Santa Clara, CA, USA). The column was washed twice with 10 mL of ultrapure water and dried with air. OTA bound to the specific antibodies within the IAC was eluted with 2 × 0.5 mL of methanol at a flow rate of 1–2 drops per second, followed by the addition of 1 mL of ultrapure water. The final eluate was collected in HPLC vials and stored at 4 °C until chromatographic analysis.

For DON, the extract was filtered through Whatman pre-folded filter paper followed by a glass microfiber filter. A 2 mL aliquot was passed through a DONtest™ IAC (VICAM, Watertown, MA, USA) using a vacuum manifold. The column was washed twice with 10 mL of ultrapure water and dried with air. DON was eluted with 1 mL of methanol, followed by 1 mL of ultrapure water. The eluate was collected in HPLC vials and stored at 4 °C until analysis.

Chromatographic analyses of OTA and DON were conducted using a Shimadzu HPLC system (Tokyo, Japan) comprising an LC-20AP pump, SIL-20A auto-sampler, FCV-200AL quaternary valve, CTO-10AS VP column oven, CBM-20A system controller, and either a fluorescence detector (RF-20A XS) for OTA or a UV–visible detector (SPD-40) for DON. Separation was achieved using a reversed-phase InertSustain C18 column (4.6 × 150 mm, 5 μm; GL Sciences, Tokyo, Japan).

For OTA, the mobile phase consisted of acetonitrile–water–acetic acid (47:51:2, v/v/v) delivered at a 1 mL/min, with the column maintained at 45 °C. Detection was performed via fluorescence at excitation/emission wavelengths of 333/460 nm. For DON, isocratic elution was achieved using a mobile phase of water–methanol (85:15, v/v) a flow rate of 1 mL/min. Detection was carried out with a UV detector at 220 nm, and the column temperature was maintained at 40 °C. The injection volume was 100 µL for both mycotoxins.

### Method validation

The analytical methods were validated in-house with respect to linearity, accuracy (recovery), precision, and sensitivity, including limits of detection (LOD) and quantification (LOQ). Linearity was assessed by constructing calibration curves for OTA and DON across their respective concentration ranges. For OTA, six calibration standards were prepared at concentrations of 0.25, 0.50, 1, 5, 10, and 20 µg/L. Similarly, standards for DON were prepared at 10, 20, 50, 100, 200, and 500 µg/L. Each standard solution was injected in triplicate, and linear regression analysis was performed by plotting peak area against analyte concentration. The coefficients of determination (*R*^2^) were calculated to assess the goodness of fit for each calibration curve.

Accuracy and precision were evaluated through recovery experiments. A representative blank matrix obtained from commercially available wheat flour tortillas was used for all validation experiments. Blank samples were spiked at two levels for each analyte: 1 and 3 µg/kg for OTA and 100 and 500 µg/kg for DON. The spiked samples were subjected to the same extraction and clean-up procedures as described above, followed by HPLC-FLD or HPLC–UV analysis. The concentrations recovered were compared with the spiked levels, and recovery percentages were calculated. Recoveries within the acceptable range of 70–120% were considered satisfactory (EC 2023a).

Precision was assessed in terms of both intra-day (repeatability) and inter-day (within-laboratory reproducibility) variability. Repeatability was determined by analysing six replicates of spiked samples at both concentration levels on the same day by a single operator. Inter-day precision was assessed by analysing spiked samples over three consecutive days by two different operators (*n* = 12). Precision was expressed as the relative standard deviation (RSD), with values ≤ 20% considered acceptable.

The LODs and LOQs were calculated based on recovery studies conducted at the lowest spiking levels (1 µg/kg for OTA and 100 µg/kg for DON). The LOD was defined as three times the standard deviation (SD) of replicate measurements, while the LOQ was defined as ten times the SD.

### Risk assessment

A deterministic approach was employed to estimate the daily intake (EDI) of OTA and DON resulting from tortilla consumption. The EDI was calculated by combining contamination levels (mean and 95th percentile, P95) with the average daily tortilla consumption, assuming a default adult body weight of 70 kg, as recommended by EFSA (2012) (Eq.1):

1 $$EDI=\frac{Contamination\;level\;(\mu g/kg) \times Consumption\;level(kg/day)}{Body\;weigh\;(kg)}$$

Due to the absence of tortilla consumption data specific to the Turkish population, two hypothetical scenarios were developed to address variability and uncertainty in dietary exposure. Scenario 1 (56.7 kg per year) and Scenario 2 (79.5 kg per year) were derived from reported urban and rural consumption levels in Mexico (Espejel-García et al. 2016).

To address left-censored data, where analyte concentrations were below the LOD, the substitution method described by EFSA (2010) was applied. Non-detect values were replaced with"zero","LOD value", and “LOD/2” to estimate lower bound (LB), upper bound (UB), and middle bound (UB) exposure levels, respectively.

For OTA, risk characterization was conducted using the MOE approach, as recommended by EFSA for genotoxic and carcinogenic compounds. The MOE was calculated by comparing the estimated exposure levels to the benchmark dose lower confidence level (BMDL_10_), which is set at 14.5 µg/kg body weight/day for OTA (EFSA 2020) (Eq.2):

2 $$MOE= \frac{BMDL10(14.5 \mu g/kg bw/day)}{EDI (\mu g/kg bw/day)}$$

An MOE below 10,000 is considered indicative of a potential health concern, whereas values above this threshold suggest a low level of risk.

In contrast, chronic dietary exposure to DON was evaluated against TDI of 1 µg/kg bw/day, as establihed by EFSA (EFSA 2013). This comparison provided a benchmark to assess the potential health risk associated with DON intake.

## Results and discussion

### Validation data

Validation data for the detection of OTA and DON in tortilla samples are presented in Table 1. Excellent linearity was achieved for both mycotoxins, with *R*^2^ of 0.9997 for OTA and 0.9999 for DON, confirming the method’s reliability in quantifying the target analytes across the tested concentration ranges (0.5–20 μg/L for OTA and 10–500 μg/L for DON). The LODs and LOQs were determined as 0.078 and 0.26 μg/kg for OTA, and 12.9 and 43 μg/kg for DON, respectively. These values were well below the European Union maximum levels (EU MLs) of 3 μg/kg for OTA (EC 2023b) and 400 μg/kg for DON (EC^ 2024^).

**Table 1:** Validation data for OTA and DON determination in tortilla

Mycotoxin	Linearity			LOD^a^ (μg/kg)	LOQ^b^ (μg/kg)	Spike level (μg/kg)	Recovery (%)	Intra-day repeatability, % RSD (*n* = 6)	Within-laboratory reproducibility, % RSD (*n* = 12)
	Range (μg/L)	Linear regression equation	*R*^2^						
OTA	0.25–20	*y* = 85,536 *x* – 2919.8	0.9997	0.078	0.26	1	93.1	4.57	5.06
						3	93.5	4.23	3.45
DON	10–500	*y* = 2154.8 *x* – 4900.5	0.9999	12.9	43.0	100	85.4	7.28	9.76
						500	88.7	6.45	7.53

*R*^2^: Coefficient of determination

^a^ LOD: Limit of detection

^b^ LOQ: Limit of quantification

Recovery rates varied with the spiking concentrations, demonstrating the method's effectiveness. OTA recoveries were 93.1% at 1 μg/kg and 93.5% at 3 μg/kg, while DON recoveries were 85.4% and 88.7% at 100 and 500 μg/kg, respectively. Intra-day repeatability (expressed as RSD) was 4.57% and 4.23% for OTA and 7.28% and 6.45% for DON at low and high spiking levels, respectively. Inter-day reproducibility (within-laboratory) showed slightly higher RSD values but remained within acceptable limits, ranging from 3.45% to 5.06% for OTA and from 7.53% to 9.76% for DON.

All results fell within the acceptable ranges for recovery (70–120%) and precision (≤ 20% RSD) as specified in the regulatory guidelines (EC 2023a), thereby confirming the accuracy, precision, and overall robustness of the analytical method.

### Occurrence of OTA and DON in tortillas

The natural occurrence and levels of OTA and DON detected in the tortilla samples are summarized in Table 2. OTA was detected in 25% of the tortilla samples, with contamination levels ranging from 0.079 to 0.930 µg/kg. The mean OTA concentration across all samples was determined to be 0.260 µg/kg. None of the OTA-positive samples exceeded the EU ML of 3 µg/kg. OTA was identified in five wheat tortillas (0.096–0.930 µg/kg), seven whole wheat tortillas (0.190–0.723 µg/kg), two whole cereal tortillas (0.079–0.433 µg/kg), four maize tortillas (0.088–0.103 µg/kg), one oat tortilla (0.361 µg/kg), and two tortillas of other varieties (0.103–0.126 µg/kg).

**Table 2:** Distribution and concentrations of OTA and DON detected in tortilla samples

Type of tortilla	Parameters	OTA	DON
Wheat tortillas (*n* = 16)	Positive samples^a^ *n* (%)	5 (31.3)	1 (6.3)
	Range (min–max, μg/kg)	0.096^b^–0.930	158
	Mean of positive samples (μg/kg)	0.355	158
	Mean value (LB–UB^c^ μg/kg)	0.134 (0.111–0.165)	16.8 (9.9–23.8)
Whole wheat tortillas (*n* = 17)	Positive samples *n* (%)	7 (41.2)	3 (17.6)
	Range (min–max, μg/kg)	0.190^b^–0.723	69–127
	Mean of positive samples (μg/kg)	0.315	95
	Mean value (LB–UB, μg/kg)	0.150 (0.130–0.176)	23 (16–29)
Whole cereal tortillas (*n* = 10)	Positive samples *n* (%)	2 (20)	2 (20)
	Range (min–max, μg/kg)	0.079^b^–0.433	13.1^b^–14.3
	Mean of positive samples (μg/kg)	0.256	13.7
	Mean value (LB–UB, μg/kg)	0.078 (0.051–0.114)	7.9 (2.8–13.1)
Maize tortillas (*n* = 13)	Positive samples *n* (%)	4 (30.8)	1 (7.7)
	Range (min–max, μg/kg)	0.088^b^–0.103^b^	33^b^
	Mean of positive samples (μg/kg)	0.095	33
	Mean value (LB–UB, μg/kg)	0.053 (0.029–0.083)	8.5 (2.5–14.4)
Oat tortillas (*n* = 10)	Positive samples *n* (%)	1 (10)	2 (20)
	Range (min–max, μg/kg)	0.361	43–75
	Mean of positive samples (μg/kg)	0.361	59
	Mean value (LB–UB, μg/kg)	0.067 (0.036–0.106)	17 (12–22)
Other varieties (*n* = 18)	Positive samples *n* (%)	2 (11.1)	–^d^
	Range (min–max, μg/kg)	0.103^b^–0.126^b^	–
	Mean of positive samples (μg/kg)	0.115	
	Mean value (LB–UB, μg/kg)	0.043 (0.013–0.082)	6.5 (0–12.9)
∑ (*n* = 84)	Positive samples *n* (%)	21 (25)	9 (10.7)
	Range (min–max, μg/kg)	0.079^b^–0.930	13.1^b^–158
	Mean of positive samples (μg/kg)	0.260	69
	Mean value (LB–UB, μg/kg)	0.091 (0.065–0.124)	13.1 (7.4–18.9)

^a^ Positive samples: mycotoxin level ≥ LOD

^b^ < LOQ

^c^ LB–UB: lower bound–upper bound. LB: results below the limit of detection were replaced with 0, UB: results below the limit of detection were replaced with the value of limit of detection

^d^ –, not detected, i.e., below LOD

In contrast, DON was found in only 10.7% of the samples, with contamination levels ranging from 13.8 to 158 µg/kg. The mean concentration of DON among the positive samples was calculated as 69 µg/kg, which remained well below the EU ML of 400 µg/kg. DON was detected in one wheat tortilla (158 µg/kg), three whole wheat tortillas (69–127 µg/kg), two multi-grain tortillas (13.1–14.3 µg/kg), two oat tortillas (43–75 µg/kg), and one maize tortilla (33 µg/kg). DON was not detected in other varieties, including chocolate, spinach, chia, protein-enriched, and spiced tortillas.

To date, studies investigating OTA and DON contamination in tortillas have been limited. No prior research has specifically addressed OTA occurrence and only limited data are available for DON. This knowledge gap is particularly noteworthy given the widespread consumption of tortillas, especially in Latin America, where maize-based varieties represent a dietary staple. Previous studies have primarily focused on AFs and FUM (Castillo-Urueta et al. 2011; Wall-Martínez et al. 2019; Garsow et al. 2024), due to their high prevalence and well-established health risks in regions where maize is commonly consumed, particularly in Africa and Latin America. In contrast, OTA and DON contamination is more commonly associated with cereals cultivated in temperate climates, which may explain the limited research attention in Latin American tortilla products. Nevertheless, considering the possible health implications, further comprehensive studies on mycotoxin contamination, including OTA and DON, in staple foods such as tortillas are warranted.

The contamination of tortillas with OTA and DON may result from multiple factors, primarily related to the contamination of raw materials, environmental conditions, and post-harvest handling practices. OTA is mainly produced by *Penicillium* and *Aspergillus* species, which thrive under warm and humid conditions. Therefore, improper storage of cereal grains such as wheat, oats, rye, and maize can promote fungal growth and subsequent OTA production. Similarly, DON is produced by *Fusarium* spices, and is typically associated with unfavourable weather conditions during the growing season, particularly in temperate climates. Consequently, the contamination of raw grains used in tortilla production may reflect the climatic conditions and agricultural practices of the regions where the crops were cultivated.

The presence of OTA and DON in cereals and cereal-based products has been extensively documented, underscoring the potential for contamination in raw materials used in tortilla production. In a previous study conducted in the Kabak laboratory, OTA was detected in 9.2% of 141 wheat samples from Turkey, with concentrations ranging from 0.1 to 3.2 µg/kg (Turksoy and Kabak 2020). In another study, OTA was recorded in 10 out of 102 wheat bread samples (9.8%), a staple food in Turkey, with concentrations reaching up to 2.83 µg/kg (mean = 0.16 µg/kg) (Golge and Kabak 2016).

In Lebanon, OTA was found in 8% of wheat flour samples, with concentrations ranging from 0.6 to 3.4 µg/kg; only one sample exceeded the ML of 3 µg/kg for wheat-derived products (Elaridi et al. 2019). In contrast, Daou et al. (2021) reported OTA in all wheat and wheat product samples analysed (*n* = 312), with concentrations ranging from 0.02 to 63.3 µg/kg. Notably, 17.6% of the samples surpassed the EU ML for OTA.

In Spain, OTA was identified in 3 out of 80 breadcrumb samples (3.8%), with concentrations ranging from 1.5 to 6.75 µg/kg (Luz et al. 2018). In contrast, no detectable levels of OTA, AFs, DON, or ZEA were reported in wheat flour samples under varying storage conditions in Italy (Annunziata et al. 2021).

With regard to DON contamination, Golge and Kabak (2020) reported its presence in 26% of wheat (58–1092 µg/kg), 13.3% of maize (313–331 µg/kg), 20% of barley (138–973 µg/kg) 6% of wheat flour (92–151 µg/kg), 35% of rice (136–256 µg/kg), 10% of pasta (49.3 µg/kg), and 20% of biscuit samples (31.2–71.3 µg/kg) in Turkey, with all values remaining below the EU MLs. No DON was detected in bulgur or wheat bread samples.

In Pakistan, Iqbal et al. (2020) identified DON in 43.4% of wheat and wheat-based products (195 out of 449 samples) and 53.3% of maize and maize-based products (144 out of 270 samples), with concentrations reaching up to 2145 and 2490 µg/kg, respectively. In Egypt, Gab-Allah et al. (2022) reported a higher prevalence of DON in maize flour (83.3%) compared to wheat flour (56%), with concentrations ranging from LOQ to 853 µg/kg (mean = 330 µg/kg) in maize flour and from LOQ to 389 µg kg⁻^1^ (mean = 180 µg/kg) in wheat flour.

Additionally, commercial maize and wheat flour samples were analysed for DON and its acetylated derivatives by Cao et al. (2021). DON was detected in 80% of maize flour and 60% of wheat flour samples, with concentrations ranging from 1.4 to 3199 µg/kg and 0.8 to 3245 µg/kg, respectively. Acetylated derivatives, including 3-acetyl-DON (3-ADON) and 15-acetyl-DON (15-ADON), were identified in 20% of maize flour samples, but none were detected in wheat flour. These findings highlight the variability in OTA and DON contamination across regions and product types, influenced by climatic conditions, storage practices, and agricultural methods.

Cross-contamination during processing represents another critical factor contributing to OTA and DON presence in tortillas. In facilities handling multiple grain types, the inadvertent mixing of contaminated batches may occur, particularly in the absence of effective cleaning protocols. Moreover, mycotoxin levels may persist or even increase during storage and transportation, especially when grains are exposed to moisture. Although nixtamalization has been shown to reduce certain mycotoxins, such as AFs (Zavala-Franco et al. 2020) and FUM (Voss et al. 2006), its efficacy against OTA and DON appears to be limited, potentially due to the chemical stability of these toxins under alkaline conditions.

The detection of OTA and DON in tortillas is of particular concern, given their chronic toxicological effects and the health risks associated with prolonged dietary exposure. The presence of multiple mycotoxins also raises the possibility of co-exposure, which may result in additive or synergistic toxic effects. This is especially important in regions such as Mexico and Central America, where tortillas constitute a major dietary component. In the present study, co-occurrence of OTA and DON was observed in three tortilla samples, two of which were whole wheat tortillas and one a maize-based tortilla. These findings emphasise the importance of simultaneous monitoring for multiple mycotoxins, as co-contamination may heighten the overall food safety risk.

### Health risk assessment of OTA and DON in tortillas

The risk associated with OTA and DON exposure through tortilla consumption was assessed under two distinct dietary scenarios. For OTA, the estimated mean exposure values in adults ranged from 1.4 to 2.7 × 10^–4^ µg/kg bw per day under Scenario 1, and from 2.0 to 3.8 × 10^–4^ µg/kg bw per day under Scenario 2. The corresponding mean MOE values were calculated as 100,744–52,809 and 71,960–37,721, respectively. At the P95 exposure level, OTA intake was estimated at 7.9 × 10^–4^ µg/kg bw per day for Scenario 1 and 0.001 µg/kg bw per day for Scenario 2, with the respective MOE values determined to be 18,353 and 13,109. As all calculated MOE values exceeded the critical threshold of 10,000, the health risk associated with OTA exposure in adults under both scenarios was considered to be low.

These values were slightly lower than those reported in a previous study, which estimated a mean OTA intake of 8.5 × 10⁻^4^ µg/kg b.w. per day from the consumption of wheat breads, a staple food in Turkey (Golge and Kabak 2016). Moreover, the estimated exposure levels from tortillas in the present study were notably lower than those reported in other regions for wheat bread consumption, including 0.0011 µg/kg bw per day in Portugal (Bento et al. 2009), 0.0016 µg/kg bw per day in Spain (Gonzalez-Osnaya et al. 2007), and as high as 0.126 µg/kg bw per day in Morocco (Zinedine et al. 2007).

In the Turkish population, OTA exposure has been primarily attributed to cereals and cereal-based products, which account for approximately 75.3% to 85.7% of total dietary intake, followed by chocolate and nuts. Within the cereal group (excluding tortillas), wheat bread has been identified as the major contributor, representing 55.4% to 69.2% of total OTA exposure. This is despite its relatively low mean OTA concentration (mean MB = 0.056 µg/kg; P95 MB = 0.085 µg/kg), and is largely attributable to its high consumption rate (2.232 kg per week) relative to other cereal-based products (Kulahi and Kabak 2020). In the EU, cereals have similarly been recognised as the main contributor to OTA exposure, accounting for approximately 50% of total intake, followed by wine (13%), coffee (10%), spices (8%), and dried fruits (3%) (Miraglia and Brera 2002). Comparable trends have been observed in the Dutch population, where cereals contributed 57% to overall OTA exposure, with smaller contributions from coffee (9%), wine (8%), and meat (8%) (Bakker and Pieters 2002). These findings consistently emphasise the dominant role of cereal-based products in dietary OTA exposure.

For DON, the estimated mean dietary exposure ranged from 0.016 to 0.042 µg/kg bw per day under Scenario 1 and from 0.023 to 0.059 µg/kg bw per day under Scenario 2. At the P95 exposure level, DON intake was estimated at 0.144 µg/kg bw per day for Scenario 1 and 0.202 µg/kg bw per day for Scenario 2. These exposure levels remained well below the tolerable daily intake (TDI) of 1 µg/kg bw per day established by EFSA (2013), suggesting minimal health concern.

In contrast to other studies, the levels observed in this assessment were considerably lower. For instance, in China, where wheat-based products are extensively consumed, mean daily exposures to DON in adults have been reported to range from 0.275 to 0.313 µg/kg bw per day, with P95 exposures reaching 3.99 to 4.20 µg/kg bw per day (Yang et al. 2021). These P95 values substantially exceed the TDI, indicating a potential health risk, as they are approximately four times higher than the established threshold. Similarly, the EFSA report (2013) covering 14 European countries documented mean DON exposures ranging from 0.17 to 0.46 µg/kg bw per day, and P95 exposures between 0.31 and 1.02 µg/kg bw per day. Bread and rolls were identified as the primary contributors to total DON exposure (30.9–64.2%), followed by fine bakery wares (10.6–34.3%), grain milling products (18.4–21.1%), and raw pasta (11.9–30.5%). These comparisons underscore the considerable regional variability in DON exposure, highlighting the relatively low levels observed in the present study and the minimal health risk associated with tortilla consumption under the evaluated scenarios.

This study provides important insights into the natural occurrence and concentrations of OTA and DON in tortillas produced in Turkey, thereby contributing to the limited body of literature on mycotoxin contamination in this widely consumed staple food. OTA was detected in 25% of the samples, with concentrations remaining well below the EU ML, while DON was present in 10.7% of samples, also within permissible levels. Co-contamination was observed in three samples, emphasising the importance of simultaneous monitoring of multiple mycotoxins due to their potential synergistic toxic effects. The data revealed product-specific variations in mycotoxin content, influenced by factors such as raw material contamination, environmental conditions, and post-harvest handling practices. OTA contamination was widespread across various tortilla types, whereas DON occurrence appeared more limited. The thermal and chemical stability of DON during processing, including nixtamalization, presents additional challenges and underscores the need for further research into both traditional and emerging processing techniques aimed at mitigating OTA and DON contamination. Overall, exposure to OTA and DON through tortilla consumption was found to pose minimal health risks under the scenarios evaluated. Nonetheless, considering the chronic health risks associated with prolonged low-level exposure to mycotoxins, particularly in populations for whom tortillas constitute a dietary staple, proactive measures are warranted. Particular attention should be given to vulnerable populations, including pregnant women, the elderly, and immunocompromised individuals, for whom even low-level chronic exposure to OTA and DON may pose heightened health risks. The increased susceptibility observed in these groups underscores the need for more conservative safety thresholds and the development of tailored risk assessment models. In line with this, measures such as stringent regulatory surveillance, improved storage conditions, and optimised processing practices should be prioritised. Moreover, future studies are recommended to focus on acetylated derivatives of DON and emerging mycotoxins such as enniatins and beauvericin, which are increasingly recognised for their relevance in cereal-based products. Future research should focus on evaluating effective mitigation strategies and investigating the interactive effects of co-occurring mycotoxins in tortillas to ensure consumer safety and protect public health.

## Data Availability

No datasets were generated or analysed during the current study.
